# Examining Sexual Harm in a Nightlife Precinct in Victoria, Australia

**DOI:** 10.1007/s10508-025-03277-1

**Published:** 2025-10-23

**Authors:** Kira M. Button, Nicholas Taylor, Kerri Coomber, Dominique de Andrade, Zara Quigg, Peter G. Miller

**Affiliations:** 1 School of Psychology, Deakin University, Geelong Waurn Ponds Campus, 75 Pigdons Road, Waurn Ponds, Geelong, VIC 3216 Australia; 2 National Drug Research Institute, Curtin University, Melbourne, Australia; 3 Griffith Criminology Institute, Griffith University, Brisbane, Australia; 4 School of Psychology, University of Queensland, Brisbane, Australia; 5 School of Public and Allied Health/Public Health Institute, Liverpool John Moores University, Liverpool, UK

**Keywords:** Sexual harm, Nightlife settings, Venue lighting, Dancefloor, Nightclub

## Abstract

Sexual harm within nightlife settings is a public health concern. This study aims to investigate the nature and prevalence of sexual harm experienced by Australian nightlife patrons and examine sociodemographic and environmental factors associated with sexual harm. Street interviews were conducted with patrons (*N* = 232, 51.3% women) in one night-time entertainment precinct in Victoria, Australia. Logistic regression analyses examined individual (e.g., pre-drinking) and venue-level (e.g., lighting) predictors of sexual harm on the night of interview. In the past three months, over half (56.6%) of women and nearly one third (31.7%) of men reported experiencing nightlife-related sexual harm. Sexual harm most frequently occurred on the dancefloor, with the most common types of harm experienced on the night of the interview and in the past three months being unsolicited sexual comments, leering, and groping. Regression analyses indicated that participants who had experienced nightlife-related sexual harm in the past three months were more likely to experience sexual harm on the night of the interview (OR 6.12), while those who visited venues with brighter lighting (self-rated) were less likely to experience sexual harm (OR 0.73). The findings suggest that sexual harm is highly prevalent in Australian nightlife settings. To reduce nightlife-related sexual harm, future interventions should consider venue lighting levels and incorporate a more substantial security presence in high-risk areas.

## Introduction

Sexual harm encompasses a broad continuum of unwanted sexual behaviors, ranging from subtle, non-physical acts to explicit and severe forms of harm (Button et al., 2024; Sardina & Ackerman, 2022). These behaviors and conduct are sometimes referred to as sexual harassment (Pina & Gannon, 2012), sexual aggression (Sanchez et al., 2019), or sexual violence (Quigg et al., 2020) in existing literature. Sexual harm is a global public health and gender equality concern that can have profound social and economic impacts on victims, bystanders, healthcare services, and communities (de Andrade et al., 2019; Whetton et al., 2021; World Health Organization, 2021). Experiencing sexual harm can result in short- and long-term consequences for individuals, including depressive symptoms, fear, damaged self-image, and self-blame (Pina & Gannon, 2012; Sigurdardottir & Halldorsdottir, 2021). A large proportion (58%) of sexual harm incidents that occur in public settings take place in nightlife venues (e.g., pubs, clubs, and bars; Mellgren et al., 2018). While nightlife venues are essential in contributing to the economy in many countries and play an important role in the social development of young adults (Aresi & Pedersen, 2016; Hadfield, 2009), the highly sexualized nature of such venues may enable and encourage sexual harm (Fileborn, 2012). Despite the substantial individual and societal ramifications, high-quality research investigating sexual harm within nightlife venues is limited, particularly within the Australian context (Quigg et al., 2020).

Prevalence rates of sexual harm within nightlife venues vary substantially across the existing literature, as studies tend to utilize different definitions and measures of sexual harm (Quigg et al., 2020). One review indicated that lifetime prevalence ranged from 6.5% (male victims of unwanted sexual contact; Tinkler et al., 2018) to 82.5% (females experiencing groping in bars; Huber & Herold, 2006; Quigg et al., 2020); however, as the review did not examine grey literature, existing Australian reports were overlooked. Patron interviews conducted in 2011–2012 in Geelong (Victoria) found that only 3% of patrons reported being involved in sexual aggression in the past three months (Miller et al., 2013a). In contrast, interviews conducted across three Queensland nightlife districts in 2016 found that 36–56% of women and 14–21% of men experienced unwanted sexual attention in the past three months (Miller et al., 2019). While this discrepancy may reflect a substantial increase in sexual harm, it more likely resulted from differences in terminology used (Hamby & Koss, 2003). Research around the measurement of sexual victimization suggests that specific behavioral terms should be utilized as opposed to general or ambiguous descriptors (Hamby & Koss, 2003).

A combination of individual, environmental, and social factors likely contributes to the occurrence of sexual harm within nightlife spaces (Quigg et al., 2020). There is a substantial body of international literature indicating that women experience significantly higher rates of nightlife-related sexual harm compared to men (e.g., Becker & Tinkler, 2015; Johnson et al., 2015). This is consistent with broader literature that suggests sexual violence disproportionately affects women (Borumandnia et al., 2020). Further, there are mixed findings regarding the relationship between age and experiences of sexual harm in nightlife settings. Some studies suggest that younger individuals are more likely to be targeted (Sanchez et al., 2019), while others have found no significant association (Baldwin et al., 2022; Hughes et al., 2008), or report that the association exists only among women (Palamar & Griffin, 2020).

Moreover, risky alcohol consumption practices such as pre-drinking and binge drinking have been associated with sexual harm victimization (Abbey, 2002; Buddie & Parks, 2003; Santos et al., 2015). For example, a Brazilian study of nightlife patrons found engaging in pre-drinking increased the likelihood of experiencing sexual harassment by 2.9 times for women (Santos et al., 2015). Alcohol use may increase the risk of sexual harm victimization by diminishing an individual’s ability to effectively evaluate how others perceive them, which may lead to misperceptions in how the target perceives the perpetrator’s intent and vice versa (Testa et al., 2000). For instance, intoxicated men are more likely to perceive indirect avoidance strategies and friendly gestures used by women as flirtatious or a sexual invitation (Abbey et al., 2001). Further, heavily intoxicated patrons showing signs of impairment may be deliberately targeted by perpetrators (Graham et al., 2014). Rates of binge drinking and pre-drinking are relatively high in Australia compared to other countries (Ferris et al., 2019; Winstock et al., 2021); therefore, these risky drinking behaviors may exacerbate the risk of sexual harm within Australian nightlife venues.

In the context of environmental factors, venue staff who participated in focus group research have suggested that having an adequate lighting level within venues could mitigate the risk of sexual harm (Powers & Leili, 2016). To date, very little research has been conducted to investigate the relationship between venue lighting level and sexual harm. However, findings from recent research on the impact of venue lighting on illicit drug use (Carlini et al., 2017), alcohol consumption (Labhart et al., 2021), and violence (Gregory et al., 2022) suggest that lighting level can impact patron behavior. One Australian policing study found that when the median lighting level of a venue is above 20 LUX, the probability of a violent incident occurring in the venue decreases to almost zero (Gregory et al., 2022). Furthermore, while music genre has been associated with nightlife-related sexual harm in prior research (Palamar & Griffin, 2020; Sanchez et al., 2019), the impact of venue noise level has not been investigated. Early nightlife observational research suggested that elevated noise levels within venues may make patrons feel more discomfort, which in turn could lead to aggression (Macintyre & Homel, 1997). However, further research is needed to determine whether venue noise and lighting level impact nightlife-related sexual harm.

Relatedly, Graham and colleagues conducted seminal observational research on sexual harm in Canadian nightlife settings (Graham et al., 2006, 2010). Key findings suggested that incidents of sexual harm typically involved a male perpetrator and female target (Graham et al., 2010). Observers also noted that intoxicated individuals were often targeted and that sexual harm frequently occurred in specific locations within venues, including the dancefloor and walkways (Graham et al., 2010). However, the generalizability of these findings may be limited due to low inter-rater reliability, the increasingly dated data collected (these studies were conducted in 2001), and differences in nightclub operations across countries (Graham et al., 2006). Nightlife settings are dynamic spaces that often change over time; for instance, nightlife settings were once characterized as male-dominated, whereas now, women play a more prominent and active role (Lindsay, 2005). As such, older literature may not accurately reflect modern nightlife environments.

A recent review found that < 10% of existing literature on nightlife-related sexual harm is from the Western Pacific Region, with the majority of research being conducted in the Americas or Europe (Quigg et al., 2020). Factors, such as social norms, drinking culture, legal drinking age, and nightclub characteristics, can differ substantially between countries (Carpenter & Dobkin, 2011; Hughes et al., 2011; Quigg et al., 2020; Winstock et al., 2021). Given the considerable differences in nightlife environments and patrons across countries, further research is needed to examine sexual harm specifically within Australian nightlife settings. Finally, literature on the prevention of sexual harm within nightlife venues is scarce, with very few evaluative studies having been conducted in the space (Button et al., 2024; Quigg et al., 2020). Developing a comprehensive understanding of the factors that contribute to sexual harm will help inform the development of evidence-based interventions in Australia.

### The Current Study

The current study aims to investigate the prevalence and types of sexual harm experienced by Australian nightlife patrons and investigate the relationship between sexual harm, gender, age, risky drinking behaviors, history of victimization, lighting, and noise level. It is hypothesized that:

1. Women will be significantly more likely to report experiencing sexual harm compared to men.
2. Participants who report pre-drinking and have a higher blood alcohol concentration (BAC) will be significantly more likely to experience sexual harm than those who did not pre-drink or have lower BAC levels.
3. Participants who attended venues they rated as having lower lighting levels will be significantly more likely to experience sexual harm than those who report higher lighting levels.

## Method

### Participants

Participants were recruited around nightlife venues in Geelong, the largest regional city in Victoria, Australia (2021 population = 271,057; Australian Bureau of Statistics, 2022). Participants were only interviewed if they were 18 years of age or older (the legal drinking age in Australia). The research assistants approached 303 individuals, with interviews being completed by 232 participants (76.1% participation rate). Participants’ ages ranged from 18 to 62 (*Mdn* = 21, IQR 19, 24). The sample consisted of 119 women, 110 men, 1 non-binary individual, and 2 individuals who did not specify their gender. Participants had been “out partying” for an average of 3.8 h (SD = 3.04) at the time of the interview.

### Procedure

The patron interviews were based on established protocols (Miller et al., 2013b, 2017) and were designed to gather a systematic random sample. Research assistants received training facilitated by the senior researcher prior to data collection. This training included guidance on recruitment procedures in nightlife settings, recognizing signs of intoxication, managing potentially aggressive behavior, and using the survey platform. Teams of four or more trained research assistants sampled participants by approaching approximately every third patron that passed by in busy thoroughfares or who was queueing for a venue. Individuals who were assessed by researchers as too intoxicated to provide informed consent were not approached for participation. To ensure the safety of the research team, a supervisor was appointed to oversee and support the research assistants during data collection.

Participants were first briefed on the aims of the research and then asked to provide verbal consent before beginning the interview. Participants were informed that their involvement was entirely voluntary and they could cease the interview at any time without consequence. Interviews were, on average, 8–10 min in length. Data were collected on iPhone and iPad devices via the offline Qualtrics Survey Application (Qualtrics, 2023). To ensure consistency, efficiency, and accuracy of data collection, and to prevent potential damage or theft of devices, researchers read the questions aloud and entered responses on the device. At the conclusion of the interview, participants undertook a breathalyzer test (Andatech Prodigy S) and were informed of their BAC reading. Participants were given an information card containing the contact details of the ethics committee and lead investigator and were informed that they could withdraw their data after the interview. No participants requested for their data to be withdrawn. Recruitment took place between 10 p.m. and 3 a.m. on Saturday nights from December 2022 until February 2023.

### Measures

The interview tool was modified from previous research (Miller et al., 2013a) and comprised of several sections, including demographics, the current night out, and experiences of nightlife-related aggression and sexual harm. The factors measured in the current study were selected based on prior empirical research, practical considerations around survey length, and areas identified as underexplored. The specific items utilized in the current study are discussed below.

#### Demographics

Age was included as a continuous variable. Participants were asked what gender they identified as. Due to the limited responses from individuals who identified as non-binary (*n* = 1), gender was included as a dichotomous variable in the logistic regression analyses (women = 0, men = 1).

#### Hour of Interview

The hour of the interview was measured as an ordinal variable. There were five one-hour categories, spanning from 22:00 to 03:00 (1 = 22:00–22:59, 2 = 23:00–23:59…).

#### Pre-drinking

Participants were asked, “Did you consume any alcohol before going out to a licensed venue tonight (e.g., in a private home or other private setting)?” Pre-drinking was included as a dichotomous variable (0 = no, 1 = yes).

#### Blood Alcohol Concentration (BAC)

BAC at the time of the interview was used to determine participants’ intoxication level and was measured as a categorical variable. In accordance with prior research, BAC was categorized into five groupings: sober (0.000 g/dl); slightly intoxicated (0.001–0.049 g/dl); moderately intoxicated (0.050–0.079 g/dl); intoxicated (0.080–0.119 g/dl); and heavily intoxicated (0.120 g/dl and above). The breathalyzer model was an Andatech Prodigy S, calibrated 6-monthly. The BAC setting was utilized, whereby the breathalyzer converted breath alcohol readings to BAC.

#### Subjective Lighting and Noise Level of Venues

Participants were asked questions about the venues they had visited so far on the night of the interview. On a ten-point Likert scale, participants were asked, “How well lit did you think this venue was?” (0 = too dark, 5 = about right, 10 = too bright) and “How noisy did you think this venue was?” (0 = too quiet, 5 = about right, 10 = too noisy). Participants could rate up to three venues that they had attended on the night. Some participants (32.8%) did not rate any venues as they had either not been to a venue yet or ceased the interview early. These participants were not included in the analysis. An average lighting level and an average noise level were produced for each participant who visited two or more venues (i.e., lower averages indicate that participants tend to visit darker and quieter venues). For those who had only been to one venue, a singular lighting and noise score was used.

#### Sexual Harm

Participants were asked, “Have you experienced any of the following in/around licensed venues: sexual comments, groping, pinching, staring/leering, sexual assault, persistent harassment/coercion, unwanted kissing, or other sexual harm?” Participants could select multiple harms if relevant. The behaviors listed were drawn from prior literature on sexual harm in nightlife settings (e.g., Anitha et al., 2021; Huber & Herold, 2006; Sanchez et al., 2019), as well as definitions provided by government and other professional bodies (e.g., Fair Work Ombudsman, 2022; Australian Human Rights Commission, 2004). Survey instructions made clear that these questions pertained to behaviors the participants viewed as unwanted or harmful.

If participants experienced any type of harm, they were also asked, “In what area in or around licensed venues did this occur?” These questions were posed in two variations: firstly, the experience of nightlife-related sexual harm on the night of the interview, and secondly, their experiences in the past three months. Dichotomous sexual harm variables (0 = no, 1 = yes) were created for both the night of the interview and the past three months. A value of ‘1’ indicated that participants had experienced one or more types of sexual harm within or around nightlife settings on the night of the interview/past three months.

### Analytic Strategy

Frequencies and descriptive statistics were used to determine prevalence rates (across demographic variables), the types of sexual harm experienced, and locations of sexual harm. Chi-square analyses were conducted to determine if there was a significant difference between sexual harm across gender and age groups. A logistic regression analysis was undertaken to determine whether patron BAC, pre-drinking, history of sexual harm, subjective venue noise, and lighting levels were significant predictors of experiencing sexual harm on the night of the interview. Assumptions were met for the logistic regression analysis, and all analyses were conducted using IBM SPSS Statistics 27.

## Results

Table 1 shows the prevalence of nightlife-related sexual harm within the sample. The chi-square test for independence demonstrated no association between gender and sexual harm on the night of the interview. However, the same statistical test examining the association between gender and sexual harm in the past 3 months revealed a statistically significant relationship, indicating that women experienced more sexual harm than men. There were no significant differences by age group.

**Table 1:** Prevalence of sexual harm by gender and age

Characteristic (n)	N answering ‘yes’	Percent	*χ*^2^	*p*
Experience of Sexual Harm on Night of Interview				
All (*n* = 223)	56	25.1		
Gender (*n* = 219)			.57	.45
Women (*n* = 116)	31	26.7		
Men (*n* = 103)	23	22.3		
Age (*n* = 221)			2.25	.52
18–21 years (*n* = 123)	31	25.2		
22–25 years (*n* = 58)	14	24.1		
26–30 years (*n* = 24)	4	16.7		
30 + years (*n* = 16)	6	37.5		
Experience of Sexual Harm in Last 3 Months				
All (*n* = 218)	99	45		
Gender (*n* = 217)			13.59	< .001
Women (*n* = 113)	64	56.6		
Men (*n* = 104)	33	31.7		
Age			3.31	.35
18–21 years (*n* = 120)	57	47.5		
22–25 years (*n* = 58)	22	37.9		
26–30 years (*n* = 24)	12	50		
30 + years (*n* = 16)	7	43.8		

Table 2 shows the frequency of each type of sexual harm and location on the night of the interview and in the previous three months. Notably, sexual harm was most frequently reported to occur on the dancefloor, near the bar, and walking between venues and was least commonly reported to occur near the toilets or in seated areas. In the past three months, the most commonly reported type of sexual harm experienced by women was unwanted sexual comments, followed by groping and staring or leering. Among men, the most frequently reported harm was staring or leering, followed by groping.

**Table 2:** Location and type of sexual harm

	Night of the interview n (%)			Past 3 months n (%)		
Sexual Harm Type	Total *n* = 56	Wome*n* n = 31	Men *n* = 23	Total *n* = 82	Wome*n* n = 56	Men *n* = 24
Staring or Leering	27 (48.2%)	15 (48.4%)	12 (52.2%)	44 (53.7%)	32 (57.1%)	12 (50%)
Sexual Comments	23 (41.1%)	15 (48.4%)	8 (34.8%)	56 (68.3%)	44 (78.6%)	10 (41.7%)
Groping	18 (32.1%)	9 (29%)	8 (34.8%)	44 (53.7%)	32 (57.1%)	11 (45.8%)
Persistent Harassment/Coercion	12 (21.4%)	7 (22.6%)	4 (17.4%)	29 (35.4%)	25 (44.6%)	3 (12.5%)
Unwanted Kissing	5 (8.9%)	2 (6.5%)	3 (13%)	15 (18.3%)	10 (17.9%)	5 (20.8%)
Pinching	5 (8.9%)	2 (6.5%)	1 (4.3%)	14 (12.2%)	11 (19.6%)	3 (12.5%)
Sexual Assault	4 (7.1%)	2 (6.5%)	2 (8.7%)	10 (11.1%)	6 (10.7%)	3 (12.5%)
Other	4 (7.1%)	1 (3.2%)	2 (8.7%)	5 (6.1%)	3 (5.4%)	2 (8.3%)

Location of Sexual Harm	Total	Women	Men	Total	Women	Men
Dancefloor	31 (55.4%)	17 (54.8%)	12 (52.2%)	54 (65.9%)	40 (71.4%)	14 (58.3%)
Near the Bar	12 (21.4%)	5 (16.1%)	6 (26.1%)	22 (26.8%)	13 (23.2%)	9 (37.5%)
Walking Between Venues	8 (14.2%)	4 (12.9%)	4 (17.4%)	11 (13.4%)	8 (14.3%)	3 (12.5%)
Smokers Area	5 (8.9%)	2 (6.5%)	3 (13%)	9 (11%)	4 (7.1%)	5 (20.8%)
Queue for Venue	3 (5.4%)	3 (9.7%)	0 (0%)	8 (9.8%)	4 (7.1%)	4 (16.7%)
Seated Area	3 (5.4%)	2 (6.5%)	0 (0%)	7 (8.5%)	4 (7.1%)	3 (12.5%)
Near Toilet Area	2 (3.6%)	1 (3.2%)	1 (4.3%)	6 (7.3%)	3 (5.4%)	3 (12.5%)
Other	4 (7.1%)	2 (6.5%)	2 (8.7%)	5 (6.1%)	3 (5.4%)	0 (0%)

Percentages total is greater than 100% as participants could select multiple responses. The total column includes two gender diverse individuals

On the night of the interview, 75.4% of the sample reported pre-drinking, and the average BAC was .082 g/dl (SD = .052 g/dl). A binary logistic regression analysis was conducted to examine the variables associated with sexual harm on the night of the interview (see Table 3). Given that the proportion of participants reporting sexual harm did not significantly differ by gender and age (see Table 1), these variables were not included in the model. The analysis revealed that the inclusion of the independent variables significantly improved the prediction of sexual harm on the night of the interview relative to the baseline model (i.e., model with no predictors), *χ*^2^(6) = 36.01, *p* < .001. Average lighting level ratings and history of sexual harm were the only significant individual predictors in the model. As lighting ratings increased by one unit (i.e., venue was brighter), the odds of experiencing sexual harm decreased by 27%. Additionally, patrons who had experienced nightlife sexual harm in the past three months were 6.12 times more likely to experience sexual harm on the night of the interview compared to those who did not have a history of experiencing sexual harm.

**Table 3:** Logistic regression analysis for sexual harm on the night of the interview

Variables	B	SE	OR	95% CI for OR		*p*
Time of interview	.11	.20	1.12	.76	1.65	.58
Pre-drinking						
No	Ref					
Yes	.48	.58	1.61	.52	4.98	.41
BAC	− 4.26	4.52	.01	.00	99.31	.35
Average self-rated venue lighting	− .32	.12	.73	.56	.92	**.007**
Average self-rated venue noise	− .13	.12	1.14	.91	1.43	.25
Sexual harm in past three months						
No	Ref					
Yes	1.81	.44	6.12	2.61	14.35	** < .001**

*n* = 144. *OR* odds ratio, *CI* confidence interval, *BAC* Blood Alcohol Concentration. Hosmer and Lemeshow test indicated acceptable goodness of fit (*p* > .05)

### Supplementary and Sensitivity Analyses

Although gender and age were not included in the primary model due to non-significant bivariate associations, a sensitivity analysis was conducted to assess whether their inclusion would improve the model estimates. The logistic regression model including gender and age remained statistically significant, χ^2^(8) = 32.19, *p* < .001, though model fit was slightly reduced compared to the original model. Neither additional variable was statistically significant (*p* > .05), and their inclusion did not meaningfully alter the direction, magnitude, or significance of the other variables in the model.

In response to prior research suggesting that pre-drinking may increase the risk of sexual harm for women (Santos et al., 2015), an additional analysis was conducted to examine whether the relationship between pre-drinking and sexual harm varied by gender. A logistic regression model including gender, pre-drinking, and their interaction term did not significantly improve prediction of sexual harm on the night of the interview compared to the baseline model, χ^2^(3) = 1.98, *p* = .58. Neither the main effects of gender (*B* = − .96, SE = .80, *p* = .23) or pre-drinking (*B* = − .18, SE = .55, *p* = .74), nor the interaction between them (*B* = 77, SE = .55, *p* = 3.8) were significant predictors of sexual harm.

## Discussion

This study aimed to explore the nature and prevalence of sexual harm experienced by Australian nightlife patrons and investigate the sociodemographic and environmental factors associated with such harm. Hypothesis one was partially supported, as women reported experiencing more sexual harm in the past three months compared to men; however, on the night of the interview, men and women experienced similar rates of sexual harm. Pre-drinking and BAC were not significantly related to experiences of sexual harm, contrary to our second hypothesis. On the night of the interview, participants who attended venues that they rated as having lower lighting were more likely to experience sexual harm than those who attended venues that they rated as being brighter, supporting the third hypothesis; however, subjective venue noise level was not a significant predictor. Finally, exploratory analyses revealed that a history of nightlife-related sexual harm was a significant predictor of experiencing sexual harm on the night of the interview.

Notably, in the three months prior to the interview, more than one in two women and almost one in three men experienced at least one form of nightlife-related sexual harm. For women, prevalence rates were comparable to previous research on the night of the interview in Australia and the USA (Baldwin et al., 2022; Johnson et al., 2015) and within the past three months in Queensland, Australia (Miller et al., 2019). In contrast, men in the current study reported higher rates of sexual harm compared to prior patron-interview research (Baldwin et al., 2022; Johnson et al., 2015). Recent international social justice movements such as ‘Me Too’ have helped reduce stigma around experiencing sexual violence (Gallagher et al., 2019; Sigurdardottir & Halldorsdottir, 2021), which may contribute to men being more likely to disclose their experiences. Interestingly, men reported similar rates of sexual harm to women on the night of the interview, yet over the past three months, women reported considerably higher rates. One possible explanation is that men may be less likely to perceive or recall certain experiences as sexual harm unless they are particularly severe or immediate. In contrast, women may be more attuned to, or affected by, a broader range of unwanted sexual behaviors, possibly due to differences in perceived threat or socialization around personal safety (Bryden & Fletcher, 2007; Laskov et al., 2022). This could explain why men's reports did not increase over time, whereas women’s did increase.

Sexual harm most commonly occurred on the dancefloor and near the bar area. These findings are consistent with results from over one thousand observations from the Safer Bars Evaluation, which suggested that physical and sexually (e.g., groping and persistent harassment) aggressive incidents most often occurred around the bar and on the dancefloor (Graham et al., 2010, 2012). Dancefloors may be a hotspot for nightlife-related aggression and sexual harm, as it is typically an area where overcrowding occurs (Graham et al., 2012). One study found that venue-level crowding was strongly associated with forced kissing and groping within Brazilian nightclubs (Sanchez et al., 2019). Overcrowding on the dancefloor likely makes it easier for people to perpetrate sexual harm and difficult to evade contact with other patrons, which can subsequently cause challenges in identifying the perpetrator (Kavanaugh & Anderson, 2009; Thompson & Cracco, 2008).

Low lighting levels may also contribute to the disproportionate occurrence of sexual harm on the dancefloor and other high-risk areas in nightlife spaces. The current study found that on the night of the interview, patrons who visited venues they rated as being brighter were significantly less likely to experience sexual harm than those who visited darker venues. Low lighting levels have been linked to aggression and crime in many contexts (Farrington & Welsh, 2002; Gregory et al., 2022; Pease, 1999). For instance, meta-analysis findings have demonstrated that improved street lighting led to a 20% reduction in recorded crime (Farrington & Welsh, 2002). Lower lighting in high-risk areas within nightlife venues may act as a facilitator of sexual harm by granting perpetrators a greater sense of anonymity (Zhong et al., 2010). Further, darker venues may also diminish the effectiveness of venue security, as they may be less able to identify and respond to incidents of sexual harm (Kavanaugh & Anderson, 2009). It should be noted that the current study relied on patrons’ perceptions of lighting within venues. In order to substantiate these findings, future research should consider utilizing observer ratings or other objective measures of venue lighting (e.g., LUX level).

Participants who had experienced nightlife-related sexual harm in the past three months were over six times more likely to experience sexual harm on the night of the interview. These findings suggest that recent experiences of sexual harassment may increase vulnerability to further incidents, indicating a potential cycle of victimization. While it was not measured in the current study, this finding may be partly explained by patrons’ frequency of nightlife visits, as individuals who attend nightlife venues more frequently are more likely to experience sexual harm (Pino & Johnson-Johns, 2009; Quigg et al., 2024). It may also be that these patrons are more inclined to attend venues where sexual harassment more frequently occurs (e.g., pop dance or electronic venues; Sanchez et al., 2019). Further, similar trends in repeat victimization have been observed among women college students (Daigle et al., 2008). While research in this area is limited, one explanation for these findings is that the psychological consequences of sexual harm (e.g., self-blame and post-traumatic stress disorder) may increase a person’s vulnerability to future victimization by diminishing their capacity to recognize risky situational cues or engage in protective strategies (Daigle et al., 2008; Messman-Moore & Long, 2003). It is important to note that such explanations are not about blaming the person who experiences sexual harm, but rather the aim is to develop an understanding of the complex interplay of factors that may contribute to the vulnerability of victimization. Recognizing such patterns may help inform policies to prevent repeat victimization and create safer nightlife spaces.

Finally, contrary to research in other countries, patron BAC and pre-drinking were not related to sexual harm on the night of the interview (Hughes et al., 2008; Santos et al., 2015). While pre-drinking has been shown to increase the risk of sexual harm, the high prevalence of pre-drinking in the sample (75%) may limit its sensitivity and reliability as a predictor. Further, the opposing findings may be partly explained by the types of harm experienced by patrons. In prior research, pre-drinking was a significant predictor of being molested (Hughes et al., 2008) and experiencing unwanted kissing or sexual intercourse (Santos et al., 2015). In the current study, however, the most common types of sexual harm experienced were non-physical (e.g., leering and sexual comments). Higher levels of intoxication may impact physical sexual harm, as the perpetrator may target individuals who have an impaired ability to recognize and respond to the physical threat. In contrast, the typical assumption that non-physical forms of sexual harm are less serious than physical forms (Abbey et al., 2004) may reduce the perceived risk for perpetrators, leading them to target individuals regardless of their intoxication level. Further, in the current study, participants with a lower intoxication level may have been more likely to notice and report incidents of non-physical sexual harm.

The use of in situ street interviews with patrons immediately during or after their nightlife experiences offers a unique and effective method for capturing real-time data on sexual harm, and the findings from this approach have important implications for prevention and response strategies. Current suggestions for sexual harm prevention have a strong focus on individual behavior change in order to prevent victimization (Quigg et al., 2020). Future prevention efforts should be multifaceted and incorporate environmental strategies and venue management techniques. For example, a greater security presence and adequate lighting could be utilized in high-risk areas in order to deter patrons from perpetrating sexual harm and enable security staff to appropriately identify and respond to incidents. Additionally, implementing guidelines for venue layout could be beneficial, as poor design choices (e.g., location of dancefloors and bars) can lead to crowding, which in turn may increase the likelihood of physical and sexually aggressive behavior occurring (Macintyre & Homel, 1997; Sanchez et al., 2019). Given the high prevalence of sexual harm and the need to mitigate the risk of re-victimization, it is important that patrons in nightlife settings are provided with appropriate resources and assistance following such incidents. As such, following reports of sexual harm, venues should be required to provide patrons with resources that address both their immediate (e.g., acute injuries) and long-term (e.g., psychological or legal supports) needs.

### Limitations

The findings from this study should be considered within the context of several key limitations. Firstly, our prevalence estimate of sexual harm on the night of the interview may be conservative, as patrons may have gone on to experience harm following the interview. Secondly, although the sample size was sufficient to detect large effects, it may have limited the ability to identify small to medium effects. As a result of funding constraints, it was not feasible to recruit a larger sample. Thirdly, as a result of inadequate participant numbers, sexual harm was measured as a binary variable, limiting the ability to determine whether different types of sexual harm have distinct predictors. In particular, there was insufficient power to separately analyze physical and non-physical forms of harm. It is also important to consider that predictors of sexual harm may be influenced not only by behavior type, but by the pervasiveness of the experience (Langhout et al., 2005). Future studies should therefore include measures of perceived severity and impact of sexual harm. Fourthly, having researchers read the questions aloud to participants may have increased the risk of social desirability bias, particularly for sensitive questions. Fifthly, the survey did not include open-ended questions, which limited the opportunity for participants to elaborate on their experiences or provide additional context. Finally, participants were recruited from a single regional city, which may limit the generalizability of findings. Future research should endeavor to recruit larger samples from a more diverse range of night-time precincts.

### Conclusion

These findings add to the limited body of evidence investigating sexual harm in nightlife settings and highlight the magnitude of sexual harm within Australian nightlife venues. The findings indicating that patrons may be more likely to experience sexual harm in darker venues warrant further investigation. Understanding environmental and venue-level factors related to sexual harm victimization could help shift the responsibility of prevention away from the individual. While individual factors are important to consider when developing safe and inclusive nightlife spaces, a multifaceted approach to sexual harm prevention should be utilized in the development and implementation of interventions.

## Data Availability

Not applicable.
